# Overweight or obese BMI is associated with earlier, but not later survival after common acute illnesses

**DOI:** 10.1186/s12877-018-0726-2

**Published:** 2018-02-06

**Authors:** Hallie C. Prescott, Virginia W. Chang

**Affiliations:** 1 Department of Internal Medicine, Institute for Healthcare Policy & Innovation, University of Michigan, Ann Arbor, MI USA; 2 VA Center for Clinical Management Research, HSR&D Center of Innovation, 2800 Plymouth Rd. North Campus Research Center. Bldg 16, Rm 341E, Ann Arbor, MI USA; 3 Department of Social and Behavioral Sciences, NYU College of Global Public Health, New York, NY USA; 4 Department of Population Health, NYU School of Medicine, New York, NY USA

**Keywords:** pneumonia, acute myocardial infarction, congestive heart failure, obesity, hospitalization, Medicare

## Abstract

**Background:**

Obesity has been associated with improved short-term mortality following common acute illness, but its relationship with longer-term mortality is unknown.

**Methods:**

Observational study of U.S. Health and Retirement Study (HRS) participants with federal health insurance (fee-for-service Medicare) coverage, hospitalized with congestive heart failure (*N* = 4287), pneumonia (*N* = 4182), or acute myocardial infarction (*N* = 2001), 1996–2012. Using cox proportional hazards models, we examined the association between overweight or obese BMI (BMI ≥ 25.0 kg/m^2^) and mortality to 5 years after hospital admission, adjusted for potential confounders measured at the same time as BMI, including age, race, sex, education, partnership status, income, wealth, and smoking status. Body mass index (BMI) was calculated from self-reported height and weight collected at the HRS survey prior to hospitalization (a median 1.1 year prior to hospitalization). The referent group was patients with a normal BMI (18.5 to < 25.0 kg/m^2^).

**Results:**

Patients were a median of 79 years old (IQR 71–85 years). The majority of patients were overweight or obese: 60.3% hospitalized for heart failure, 51.5% for pneumonia, and 61.6% for acute myocardial infarction. Overweight or obese BMI was associated with lower mortality at 1 year after hospitalization for congestive heart failure, pneumonia, and acute myocardial infarction—with adjusted hazard ratios of 0.68 (95% CI 0.59–0.79), 0.74 (95% CI: 0.64–0.84), and 0.65 (95%CI: 0.53–0.80), respectively. Among participants who lived to one year, however, subsequent survival was similar between patients with normal versus overweight/obese BMI.

**Conclusions:**

In older Americans, overweight or obese BMI was associated with improved survival following hospitalization for congestive heart failure, pneumonia, and acute myocardial infarction. This association, however, is limited to the shorter-term. Conditional on surviving to one year, we did not observe a survival advantage associated with excess weight.

**Electronic supplementary material:**

The online version of this article (10.1186/s12877-018-0726-2) contains supplementary material, which is available to authorized users.

## Background

The impact of obesity on survival from acute medical illness remains a source of debate. While obesity is associated with worse health-related outcomes overall [1], it is often associated with a short-term (e.g. in-hospital or 30-day) survival advantage after acute decompensated congestive heart failure (CHF) [2], pneumonia [3–5], acute myocardial infarction (AMI) [6], and hospitalizations for critical illness in general—the so-called “obesity paradox”. Far less is known, however, about the association of obesity with longer-term survival. While a limited number of studies have included an extended follow-up period, they have not considered the risk of mortality conditional on surviving the shorter-term [7–9]. We hypothesized that obesity may offer short-term protection but, after surviving an initial window, may still be hazardous in the longer term given the strong association of obesity with negative health outcomes [1, 10, 11].

Recent meta-analyses and large observations studies have suggested that a body mass index of 25 to 35 appears to be protective in older patients [12, 13], leading some to argue that current weight control guidelines targeting a normal body mass index (18.5 to 25) may be harmful to older patients [14]. However, the obesity paradox may simply be the result of confounding and bias in epidemiological studies [15], explained by unmeasured differences in patients across BMI categories. Indeed, after careful control for potential confounders, a recent study found the risk of mortality increases steeply with BMIs of 27 and higher [16]. Understanding the long-term association of obesity on survival after acute hospitalization is important to guiding weight control recommendations.

Furthermore, most prior work examining the association of obesity with survival after acute hospitalization is of variable quality and limited generalizability [17]. Much of the existing data is single-center studies, often post hoc analyses of clinical databases or randomized clinical trials with limited ability to adjust for potential confounders and exclusion of large numbers of patients with missing weight [17]. Patients’ weights are generally obtained after hospital admission [17, 18], when they may be biased by acute fluid shifts. Indeed, this practice may misclassify body mass index (BMI) category in as many as 20% of patients [19]. Furthermore, many studies examine in-hospital mortality, which may be biased by discharge to nursing and long-term acute care facilities [20]. Discharge practices could plausibly be different for obese patients, who typically require more intensive nursing care for a given level of illness.

Given the high prevalence of obesity (34.6% of US adults aged 65 years and older [21]) and limited data on longer term survival after acute hospitalization, we sought to examine the association of overweight or obese BMI on longer-term survival following three common acute illnesses—congestive heart failure, acute myocardial infarction, and pneumonia—in a national sample with long-term mortality data and height and weight measurements collected prior to hospitalization. These hospitalization diagnoses accounts for 6% of US hospitalizations and 8.4% of US hospitals costs [22].

## Methods

### Study population

We studied participants in the nationally representative US Health and Retirement Study (HRS), a multistage probability sample of households that is linked to Medicare Claims [23]. Medicare is a federal health insurance program available to all US citizens ≥65 years, as well as to select younger individuals. Consent rate for Medicare records in about 80% [23]. Since the HRS cohort began in 1992, over 37,000 adults aged 51 years and older in 23,000 households have been enrolled [23]. The sociodemographic and racial distribution is broadly representative of the older US population [24–26]. The cohort is interviewed every two years, with a follow-up rate consistently over 90% [23]. Survey questions focus on wealth, health, cognition, and employment [23]. Patients provided informed consent for enrollment and again for Medicare linkage.

We used Medicare claims from 1996 to 2012, and studied all persons with a hospitalization for heart failure (CHF), pneumonia, or acute myocardial infarction (AMI) following their baseline interview. We used validated approaches to identify hospitalizations for CHF, pneumonia, and AMI by ICD-9-CM codes in linked Medicare claims (see Additional file 1: Table S1 for complete description). We followed patients until death or date of administrative censor (December 31, 2014).

### Exposures and outcomes

We sought to examine (1) the association of excess body mass index with mortality following common acute illnesses, and (2) time variation in the association. Date of death was determined from the National Death Index and confirmed by HRS interviewers and the Medicare Denominator File.

The primary exposure was overweight or obese body mass index (BMI) per World Health Organization definition (BMI ≥25.0 kg/m^2^). The reference was normal body mass (BMI 18.5 to < 25.0). In initial analyses (presented in the online supplement), we confirmed similar associations for overweight (≥25.0 to < 30.0 kg/m^2^), obese (≥30.0 to < 35.0 kg/m^2^), and severely obese (≥35.0 kg/m^2^) BMI categories relative to normal BMI, so collapsed these categories for statistical efficiency. In later analyses (also presented in the online supplement), we considered alternate and referent groups of BMI 20.0 to < 25.0 kg/m^2^ and BMI 22.0 to < 25.0 kg/m^2^.

We calculated BMI from self-reported height and weight collected at the HRS survey prior to hospitalization using the equation: BMI (kg/m^2^) = weight (kg)/height^2^ (m^2^). Self-reported height and weight have previously been validated against physical measures in the HRS [27]. Average reporting errors were small: 1–2% for height and 1–3% for weight [27]. Because HRS surveys occur biennially and are random in relation to hospitalization, there was a variable time lag between baseline weight measurement and hospitalization: CHF, median 1.1 years (IQR 0.6, 1.7); pneumonia, median 1.1 years (IQR 0.6, 1.7); AMI, median 1.0 year (IQR 0.5, 1.5).

### Covariates

We adjusted for age, sex, race/ethnicity, education, marital status, household wealth, household income, smoking status (current, former, never), admission year, and number of hospitalizations, which were abstracted from HRS surveys and linked Medicare claims. These covariates were all measured at the same time as BMI. Wealth and income were standardized to 2010 U.S. dollars using the Annual Gross Domestic Product Price Index [28], then converted to categorical variables in which category one was negative or zero assets (or income) and categories 2 through 5 were quartiles of positive assets (or income) for the entire HRS cohort. Household wealth and income were abstracted from “RAND enhanced fat file” [29], which estimates total household wealth and income using all available survey data. Giving this low rate of missingness (< 4%), we used list-wide deletion for patients with missing data. We did not adjust for medical co-morbidities in the primary analysis, as these may be viewed as mediators (not confounders) of the association between obesity and mortality, but include select co-morbidities in sensitivity analyses described below.

### Primary statistical analyses

We performed a series of cox proportional hazards models—separately for the CHF, pneumonia, and AMI cohorts—predicting mortality at several time-points following hospital admission: (1) in-hospital mortality; (2) 30-day mortality; (3) 90-day mortality; (4) 1-year mortality; and (5) 5-year mortality. We examined overall mortality at each of these time points, as well as the mortality conditional on survival to the prior time point. For patients with multiple eligible hospitalizations, we included each hospitalization in the primary analyses and accounted for clustering of hospitalizations within people using clustered robust standard errors [30]. Sensitivity analyses restricting to one hospitalization are described below. We did not adjust for clustering by household since fewer than 5% of households had more than one individual with either CHF, pneumonia, or AMI hospitalization.

### Sensitivity analyses

We performed multiple sensitivity analyses. In the first sensitivity analysis, we performed a cox proportional hazards model adjusting for the same potential confounders as in the primary analysis, but excluded patients with declining or unknown BMI trajectories prior to hospitalization. We did this sensitivity analysis because normal BMI may be confounded by illness-associated weight loss, which is indicative of poor health and increased risk for death [31]. For this analysis, we defined declining BMI trajectory as a decline in BMI by 1.0 or more between the two HRS surveys immediately preceding hospitalization.

In the second sensitivity analysis, we performed a cox proportional hazards model with adjustment for severity of illness (acute organ failures and intensive care use) in addition to the covariates included in the primary analysis. We did this sensitivity analysis because the threshold for hospitalization may vary by BMI, such that overweight and obese patients are more likely to be hospitalized with a lower severity of illness.

In the third sensitivity analysis, we performed a multivariable logistic regression model. In addition to testing the robustness of our findings to an alternate modeling approach, the multivariable logistic regression also facilitated calculating adjusted rates of mortality by BMI category.

In the fourth sensitivity analysis, we performed a cox proportional hazards identical to the primary analysis, but only one randomly selected hospitalization per person. This approach prevents multiple observations per person. We examined a randomly selected, rather than first, hospitalization for each person because we felt that first hospitalizations would not necessarily be representative of all CHF, pneumonia, or AMI hospitalizations.

In the fifth sensitivity analysis, we matched patients with normal and overweight or obesity one-to-one by age and sex. We then performed a cox proportional hazards model on matched patients, adjusting for the same potential confounders as in the primary analysis. We did this sensitivity analysis because the wide imbalance in age between normal and overweight or obesity patients may increase risk for residual confounding.

In the sixth sensitivity analysis, we adjusted for pre-morbid functional disability (count of limitations of activities and instrumental activities of daily living) and select comorbidities associated with weight loss (metastatic cancer, chronic obstructive pulmonary disease, congestive heart failure, dementia, and cerebrovascular disease) that may confound the relationship between obesity and mortality.

All analyses were conducted with Stata MP software version 14 (StataCorp, College Station, TX). We used two-sided significance testing and considered a *p* value less than 0.05 to be significant. The University of Michigan Institutional Review Board approved this study.

## Results

### CHF, pneumonia, and AMI hospitalizations

We identified 4287 CHF hospitalizations, 4182 pneumonia hospitalizations, and 2001 AMI hospitalizations for inclusion in the study (Additional file 1: Figure S1, Table 1). The cohorts were elderly (median age 77–79) and predominantly white (75–84%), with multiple medical co-morbidities (median weighted Charlson Index 3–4). Median BMI was 25–26 across the cohorts, and 52%–62% of subjects were overweight or obese. Overall 30-day mortality was 10.2% (95% CI: 9.3%–11.1%), 17.4% (95% CI: 16.2%–18.6%), and 15.6% (95% CI: 14.0%–17.3%) in the CHF, pneumonia, and AMI cohorts, respectively. 1-year mortality was 40.1% (38.6%–41.6%), 40.9% (39.4%–42.4%), and 31.9% (29.9%–34.0%).

**Table 1:** Characteristics of patients and hospitalizations, by cohort

Patient and Hospitalization Characteristics	Cohort		
	CHF *N* = 4287	Pneumonia *N* = 4182	Acute MI *N* = 2001
Age (years), median (IQR)	79 (72–86)	79 (71–85)	77 (71–84)
Male, *N* (%)	1841 (42.9%)	2007 (48.0%)	1051 (52.5%)
Race, *N* (%)			
White/Caucasian	3225 (75.2%)	3417 (81.7%)	1670 (83.5%)
Black/African American	934 (21.8%)	628 (15.0%)	258 (12.9%)
Other	128 (3.0%)	137 (3.3%)	73 (3.7%)
Married/partnered, *N* (%)	1957 (45.7%)	1967 (47.0%)	1103 (55.1%)
Wealth, *N* (%)			
Net negative or zero	592 (13.8%)	555 (13.3%)	208 (10.4%)
Quartile 1	1478 (34.5%)	1379 (33.0%)	559 (27.9%)
Quartile 2	1023 (23.9%)	1013 (24.2%)	463 (23.1%)
Quartile 3	682 (15.9%)	688 (16.5%)	432 (21.6%)
Quartile 4	512 (11.9%)	547 (13.1%)	339 (16.9%)
Income, *N* (%)			
None	1633 (38.1%)	1604 (38.4%)	684 (34.2%)
Quartile 1	1467 (34.2%)	1385 (33.1%)	632 (31.6%)
Quartile 2	831 (19.4%)	753 (18.0%)	399 (19.9%)
Quartile 3	257 (6.0%)	330 (7.9%)	219 (10.9%)
Quartile 4	99 (2.3%)	110 (2.6%)	67 (3.4%)
Body Mass Index, *N* (%)			
Underweight	165 (3.9%)	294 (7.0%)	71 (3.6%)
Normal	1538 (35.9%)	1736 (41.5%)	698 (34.9%)
Overweight/obese	2584 (60.3%)	2152 (51.5%)	1232 (61.6%)
Body mass index, median (IQR)	26 (23–30)	25 (22–29)	26 (23–29)
Co-morbidities			
Weighted Charlson Index, median (IQR)	4 (3–6)	3 (2–5)	3 (2–5)
Heart Failure, N(%)	4278 (100%)	2080 (49.7%)	1106 (55.3%)
Dementia, N (%)	303 (7.1%)	533 (12.8%)	111 (5.6%)
Metastatic Cancer, *N* (%)	86 (2.0%)	206 (4.9%)	29 (1.5%)
Smoking Status			
Current, N (%)	473 (11.0%)	601 (14.4%)	284 (14.2%)
Former, N (%)	2167 (50.7%)	2263 (54.1%)	970 (48.5%)
Never, N (%)	1647 (38.4%)	1318 (31.5%)	747 (37.3%)
Hospital Length of Stay (days), median (IQR)	5 (3–7)	5 (3–8)	5 (3–8)
Used Intensive Care, *N* (%)	974 (22.7%)	849 (20.3%)	1158 (57.9%)
Mortality, *N* (%)			
In-hospital	191 (4.5%)	455 (10.8%)	200 (10.0%)
30 days	437 (10.2%)	727 (17.4%)	312 (15.6%)
90 days	860 (20.1%)	1072 (25.6%)	411 (20.5%)
1 year	1720 (40.1%)	1710 (40.9%)	639 (31.9%)

Compared to patients with normal BMI, Patients with overweight or obese BMI were younger, less wealthy, and more likely to be male, married or partnered, and Black/African American (Additional file 1: Tables S2, Additional file 1: Table S3, Additional file 1: Table S4). In unadjusted comparisons, 30-day, 90-day, and 1-year mortality were lower in overweight and obese patients (*p* < 0.001 for each cohort). For example, 1-year mortality was 48.1% versus 33.8% after CHF hospitalization; 45.2% versus 34.8% after pneumonia hospitalizations; and 39.7% versus 25.6% after AMI hospitalization. Underweight patients had the highest 1-year mortality: 64.9%, 59.9%, and 66.2% following CHF, pneumonia, and AMI hospitalizations, respectively.

### Association of Overweight or obese BMI with mortality

In multivariable cox regression accounting for potential confounders, overweight or obese BMI was independently associated with a lower hazard of 90-day, 1-year, and 5-year mortality following congestive heart failure, pneumonia, and acute myocardial infarction hospitalizations relative to a normal BMI (18.5 to < 25.0 kg/m^2^), *p* < 0.05 for each association (Table 2). Overweight or obese BMI was also associated with lower hazard of in-hospital and 30-day mortality following CHF and pneumonia hospitalizations.

**Table 2:** Association of Overweight or Obese BMI with Mortality, by Time-Period

	Time-Period				
Type of Hospitalization	In-hospital aHR (95% CI)	30-Day aHR (95% CI)	90-Day aHR (95% CI)	1-Year aHR (95% CI)	5-Year aHR (95% CI)
Congestive Heart Failure	**0.54** (0.39–0.75)	**0.75** (0.61–0.93)	**0.69** (0.59–0.82)	**0.68** (0.59–0.79)	**0.79** (0.70–0.90)
Pneumonia	**0.75** (0.61–0.92)	**0.69** (0.58–0.82)	**0.70** (0.60–0.81)	**0.74** (0.64–0.85)	**0.82** (0.73–0.91)
Acute Myocardial Infarction	0.81 (0.56–1.15)	**0.77** (0.59–1.00)	**0.78** (0.61–1.00)	**0.65** (0.53–0.80)	**0.74** (0.63–0.87)

Adjusted hazard ratios reflect the odds of mortality during any day from hospital admission to the end-point. The referent group in BMI 18.5 to < 25.0 kg/m^2^. The models adjust for age, sex, race, marital status, education, smoking status, admission year, number of hospitalizations, household wealth, and household income

Bolded numbers are statistically significant, *p* < 0.05

### Association of Overweight or obese BMI with mortality, conditional on survival to 1 year

Among patients who survived to 1 year following acute illness, adjusted hazards of 5-year mortality were indistinguishable between patients with overweight or obese BMI versus patients normal BMI: adjusted HR for 5-year mortality 0.98 (95% CI: 0.84,1.14) in CHF, 0.92 (95% CI: 0.81, 1.05) in pneumonia, and 0.90 (0.71, 1.13) in AMI survivors (Table 3), *p* > 0.05 for all associations.

**Table 3:** Association of overweight or obese BMI with conditional mortality

	Time-Period			
Type of Hospitalization	30-Day aHR (95% CI)	90-Day aHR (95% CI)	1-Year aHR (95% CI)	5-Year aHR (95% CI)
Congestive Heart Failure	0.83 (0.64–1.09)	**0.68** (0.54–0.85)	**0.70** (0.57–0.85)	0.98 (0.84–1.14)
Pneumonia	**0.60** (0.46–0.78)	**0.74** (0.58–0.94)	**0.80** (0.66–0.97)	0.92 (0.81–1.05)
Acute Myocardial Infarction	**0.60** **(0.38–0.95)**	0.65 (0.40–1.05)	**0.53** (0.38–0.72)	0.90 (0.71–1.13)

Adjusted hazard ratios reflect odds of mortality for the time-period, conditional on survival to the prior time-period. For example, 30-day mortality is the hazard ratio for 30-day mortality, conditional survival to hospital discharge; 90-day mortality is the hazard ratio of 90-day mortality, conditional survival to 30 days. The referent group in BMI 18.5 to < 25.0 kg/m^2^. The models adjust for age, sex, race, marital status, education, smoking status, admission year, number of hospitalizations, household wealth, and household income

Bolded numbers are statistically significant, *p* < 0.05

### Sensitivity analyses

**Fig. 1:**
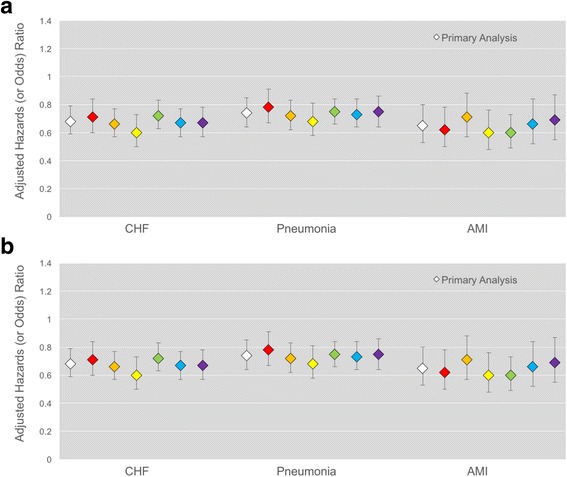
Adjusted Hazard (and Odds) Ratios for Mortality in Primary and Sensitivity Analyses Panel **a** shows 1-year mortality results. Panel **b** shows 5-year mortality, conditional on survival to one year. The adjusted hazard ratio of overweight or obese BMI, relative to normal BMI (18.5 to < 25.0 kg/m^2^) is shown. The primary analysis is in white, while sensitivity analyses are shown in color: (1, red) excluding patients with unknown BMI trajectory or declining BMI trajectory prior to hospitalization; (2, orange) adjusting for acute illness severity; (3, yellow) logistic regression model; (4, green) include just one randomly selected hospitalization per person; (5, blue) analysis of age- and sex-matched pairs; and (6, purple) adjustment for pre-morbid disability and select co-morbidities. All models adjust for age, sex, race, marital status, education, smoking status, admission year, number of hospitalizations, household wealth, and household income. Error bars represent the 95% CI for the hazard (or odds) ratio. A hazard or odds ratio of 1.0 indicates no association. A hazard ratio > 1.0 represents a positive association (overweight or obese BMI is associated with greater mortality), while a hazard ratio < 1.0 represents a negative association (overweight or obese BMI is associated with lower mortality)

The association between overweight or obese BMI and lower 1-year mortality persisted in each of the six sensitivity analyses and for all three cohorts (Fig. 1**,** Additional file 1: Table S5).

In multivariable logistic regression, adjusted 1-year mortality following CHF hospitalization was 34.8% (95% CI: 32.3%, 37.3%) in overweight and obese patients versus 46.3% (95% CI: 42.8%, 49.8%) in normal weight patients, an absolute decline of 11.5% (95% CI: 7.1%, 15.8%) *p* < 0.001. Adjusted 1-year mortality was 35.7% (95% CI: 33.4%, 38.0%) versus 44.2% (95% CI: 41.4%, 47.0%) following pneumonia hospitalizations, an absolute decline of 8.5% (95% CI: 4.8%, 12.2%) *p* < 0.001. Adjusted 1-year mortality was 27.0% (95% CI: 24.2%, 29.8%) versus 37.4% (95% CI: 33.4%, 41.3%) following AMI hospitalizations, an absolute decline of 10.4% (5.4%, 15.3%) *p* < 0.001. There was no significant association detected between overweight or obesity and 5-year survival, among patients who survive to one-year, for all sensitivity analyses.

Results were also similar when considering more granular categories of BMI (Additional file 1: Table S6). For example, adjusted HR for 1-year mortality after CHF hospitalization was 0.66 (95% CI: 0.56, 0.79), 0.71 (0.57, 0.89), and 0.70 (0.54, 0.91) among overweight, obese, and severely obese BMI, respectively. Adjusted HR for 5-year mortality, conditional on survival to 1 year after CHF was 0.95 (95% CI: 0.80, 1.13), 1.09 (0.89, 1.34), and 0.90 (0.66, 1.20) among overweight, obese, and severely obese BMI, respectively.

Results were also similar when using BMI 20.0 to < 25.0 kg/m^2^ as the referent group (Additional file 1: Table S7). Adjusted HR for 1-year mortality were 0.70 (95% CI: 0.60, 0.82), 0.77 (95% CI: 0.67, 0.89), and 0.67 (95% CI: 0.53, 0.85) after CHF, pneumonia, and AMI hospitalization, respectively. Adjusted HR for 5-year mortality, conditional on survival to 1 year, were 1.03 (95% CI: 0.88, 1.21), 0.98 (0.85, 1.13), and 0.89 (0.71, 1.13) after CHF, pneumonia, and AMI hospitalization, respectively. However, when using BMI 22.0 to < 25.0 kg/m^2^ as the referent group, the protective association of overweight and obese BMIs after CHF and pneumonia hospitalization was attenuated; adjusted HRs for 1-year mortality were 0.74 (95% CI: 0.62, 0.88) and 0.85 (95% CI: 0.71, 1.00) after CHF and pneumonia hospitalizations, respectively.

## Discussion

In this national sample of older Americans, we have shown that overweight or obese BMI is common among patients hospitalized with CHF, pneumonia, and AMI. Over half of all patients in this study had a BMI greater than 25. We found that overweight or obese BMIs were independently associated with decreased short-term mortality up to one year following hospitalization for CHF, pneumonia, and AMI, relative to normal BMI (18.5 to < 25.0 kg/m^2^). Adjusted rates of 1-year mortality were about 10% lower in overweight and obese patients, relative to normal weight patients, across each of the three cohorts. Our findings were robust to several sensitivity analyses, including analyses that accounted for BMI trajectory prior to hospitalization and severity of illness during hospitalization. They were also robust to direct standardization for age and sex in a matched analysis. The protective association of overweight and obese BMI was slightly attenuated when using a referent group of BMI 22.0 to < 25.0 kg/m^2^, suggesting that some – but not all – of the protective association may be due to increased risk of mortality for patients with low-normal BMI.

Importantly, we find that the protective effect of overweight or obese BMI does not extend into the longer term. Among patients hospitalized with CHF, pneumonia, and AMI who survived to one year, the association between overweight or obese BMI and 5-year mortality was not statistically significant in any of our analyses.

While our findings are consistent with several prior studies showing that obesity is associated with decreased in-hospital and short-term mortality following acute medical illnesses [2, 6, 18], less is known about whether such a potential advantage would extend into longer-term mortality. While there are some studies that examined longer-term survival after admission, they did not model the risk of mortality conditional on surviving the shorter-term [7–9]. Data on longer-term mortality is of central importance to the evaluation of clinical guidelines for weight management. For example, obesity may be protective against in-hospital mortality but, conditional on surviving the short term, still be hazardous for longer-term mortality. To our knowledge, this study is among the first to examine conditional, longer-term survival.

There are several biological hypotheses for why obesity may be protective during acute illness. First, while obesity is associated with greater inflammation during times of health, it also modulates the pro-inflammatory response to acute illness. In patients with acute lung injury, obesity is associated with lower levels of pro-inflammatory cytokines [32]. Obese patients have a decreased catecholamine response to stress [33]. Furthermore, adipose tissue serves as a storage reservoir for M2 macrophages, which have a protective anti-inflammatory phenotype during critical illness [34]. A recent prospective study of pneumonia patients by Singanayagam, et al., however, found that obese patients had better 30-day survival despite higher rates of sepsis and higher levels of c-reactive protein (markers of greater systemic inflammation) [5]. Moreover, Braun,et al. have recently reported improved survival in obese patients with community-acquired pneumonia, despite similar levels of c-reactive protein, white blood cell count, and procalcitonin [7]. Together, these studies suggest that alternative or additional mechanisms are likely to be involved in the protective effect.

Second, obesity may be associated with improved host response to bacterial infection. Leptin is produced by adipose tissue, modulates the immune response, and tightly correlates with BMI [35]. Leptin-deficient mice die when exposed to bacterial challenge [35].

Third, obese patients may be better poised to tolerate the catabolic stress associated with critical illness. Herridge, et al. found that patients surviving acute respiratory distress syndrome lost an average 18% of their body weight during hospitalization, and many did not return to their baseline weight by one year of follow-up [36]. This degree of acute weight loss is likely to be better tolerated by patients with a higher BMI.

Lastly, it is possible that the association it not due to a protective effect of overweight or obesity, but rather, is due to differences in the clinical presentation and/or treatment in overweight and obese patients. For example, obesity is associated with lower pulmonary reserve [37], so obese patients may become symptomatic and present earlier in their course or heart failure or pneumonia. In acute myocardial infarction, obese patients are less likely to have extensive coronary disease and left ventricular dysfunction [38]. Obese patients may also get more aggressive acute treatment, especially in cardiovascular disease, where obesity itself is viewed as a major risk factor. In two studies of patients admitted for ischemic heart disease, obese patients were more likely to receive guideline recommended therapy and undergo revascularization procedures, after adjusting for clinical risk [39, 40].

Again, we find that the protective association of excess weight is short-term and diminishes beyond one year, which is consistent with the potential biological mechanisms of benefit. Obesity may help blunt an acute inflammatory response, mediate host response to infection, and provide resilience during catabolic stress, but once these threats have resolved, obesity may cease to be protective. Thus, our findings suggest that weight control guidelines should not be reversed, as any survival benefit associated with obesity is short-term. Moreover, despite our sensitivity analyses, any residual confounding due to weight loss from severe illness is more likely to manifest in short-term mortality.

There are several limitations to our study. First, we used self-reported height and weight, which may contribute to underestimation of BMI. However, a prior analysis of the HRS cohort suggests that the magnitude of reporting errors is small [27]. Additional measures of adiposity (e.g. bioelectrical impedance, waist circumference)—which may better reflect fat mass and distribution—were not available. Secondly, CHF, pneumonia, and AMI hospitalizations were identified by ICD-9-CM codes in Medicare claims. While we used commonly employed and validated definitions, there still may be misspecification in both directions. Third, while we were able to adjust for many potential confounders not typically available in clinical or administrative data sources (e.g. household wealth and education), we could not adjust for differences in illness severity on hospital presentation beyond the crude measures of acute organ dysfunction and intensive care use. We were also unable to adjust for differences in treatment. Fourth, because HRS surveys pre-dated hospitalization by an average of one year, dynamic variables such as weight and number of functional limitations may not precisely reflect the patient’s status at the time of hospital admission. Weight at the time of admission, however, is more likely to reflect an acute fluid imbalance for some of our conditions. Fifth, because our data are from 1996 to 2012, they may not accurately represent current Medicare beneficiaries.

## Conclusions

In older Americans, overweight or obese BMI was associated with improved survival to 1 year following hospitalization for congestive heart failure, pneumonia, and acute myocardial infarction. Conditional on surviving to one year after admission, however, there is no survival advantage to 5 years associated with overweight or obese BMI.

## Additional file

Additional file 1: Online Supplement; Contains supplemental **Tables S1-S7** and supplemental **Figure S1**. (PDF 376 kb)

## Abbreviations

AMI: Acute myocardial infarction
BMI: body mass index
CHF: congestive heart failure
HRS: Health & Retirement Study
ICD-9-CM: International Classification of Diseases, 9th Edition, Clinical Modification
ICU: Intensive Care Unit
US: United States
