# Supplementary material for: Overweight or obese BMI is associated with earlier, but not later survival after common acute illnesses
Source: BMC Geriatr. 2018 Feb 6;18:42. doi: 10.1186/s12877-018-0726-2 (PMC5801673; doi:10.1186/s12877-018-0726-2)
Supplement: Additional file 1: — Online Supplement; Contains supplemental Tables S1-S7 and supplemental Figure S1. (PDF 376 kb) [file 12877_2018_726_MOESM1_ESM.pdf]

Additional files

Manuscript Title: Overweight and obese BMI is associated with earlier, but not later survival after common acute illnesses

Table S1: Cohort Definitions Page 2

Figure S1: Cohort Selection and Exclusions Page 3

Table S2: Characteristics of CHF Patients and Hospitalizations, by BMI Page 4

Table S3: Characteristics of Pneumonia Patients and Hospitalizations, by BMI Page 5

Table S4: Characteristics of AMI Patients and Hospitalizations, by BMI Page 6

Table S5: Numbers and Adjusted Hazards (or Odds) Ratios for Mortality in Primary and Sensitivity Analyses Page 7

Table S6: Adjusted Hazard Ratios from Primary Analysis, by overweight, obese, and severely obese BMI Page 9

Table S7: Adjusted Hazard Ratios from Primary Analysis, with referent BMI 20.0 to <25.0, and BMI 22.0 to <25.0 Page 10

Table S1: Cohort Definitions

Cohort	Definition
Congestive Heart Failure Exacerbation[1]	Principal ICD-9-CM code for 402.01, 402.11, 402.91, 404.01, 404.03, 404.11, 404.13, 404.91, 404.93, or 428.x
Acute Myocardial Infarction[2,3]	Principal ICD-9-CM code for 410.x with length of stay >1 day or in-patient death.
Pneumonia[4,5]	Principal ICD-9-CM code for pneumonia (480.0-480.3, 480.8, 480.9, 481, 482.1, 482.2, 482.30-482.32, 482.39-482.41, 482.49, 482.81-482.84, 482.89, 482.9, 483.0, 483.1, 483.8, 485, 486, 487.0) or principal ICD-9-CM code for sepsis (038.0-038.9, 785.52, 995.91, 995.92) or respiratory failure (518.81, 518.82, 518.84, 799.1) with a secondary code for pneumonia.

Figure S1: Cohort Section and Exclusions

Of 82,614 total Medicare hospitalizations among HRS participants, 5,078, 4,945, and 2,513 were for CHF, Pneumonia, and AMI, respectively. We excluded hospitalizations without prior data on weight or any data on height (14-17% across cohorts). We then excluded patients with missing data for age, gender, education, or marital status (<4% for each cohort).

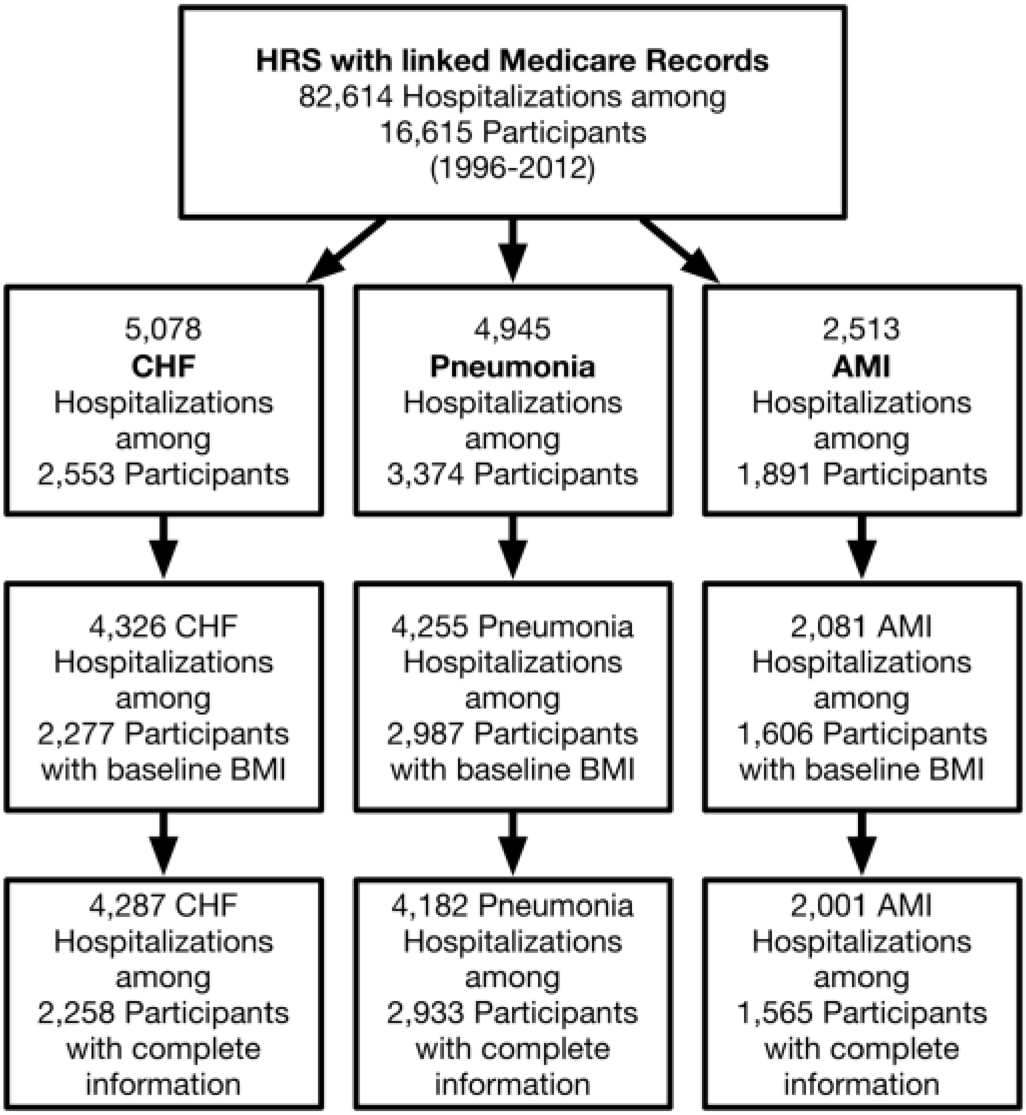

Table S2: Characteristics of CHF Patients and Hospitalizations, by BMI

	Normal BMI N=1,538	Overweight or Obese BMI N=2,584	*p*-value
Age (years), median (IQR)	82 (76-88)	76 (70-83)	<0.001
Male, N(%)	636 (41.4%)	1,178 (45.9%)	0.008
Race, N(%)			<0.001
White/Caucasian	1,250 (81.3%)	1,845 (71.4%)	
Black/African American	249 (16.2%)	653 (25.3%)	
Other	39 (2.5%)	86 (3.3%)	
Married/Partnered, N(%)	642 (41.7%)	1,273 (49.3%)	<0.001
Wealth, N(%)			<0.001
Net neg. or zero	170 (11.1%)	397 (15.4%)	
Quartile 1	460 (29.9%)	954 (36.9%)	
Quartile 2	411 (26.7%)	574 (22.2%)	
Quartile 3	283 (18.4%)	376 (14.6%)	
Quartile 4	214 (13.9%)	283 (11.0%)	
Income, N(%)			0.001
None	637 (41.4%)	934 (36.2%)	
Quartile 1	514 (33.4%)	886 (34.3%)	
Quartile 2	278 (18.1%)	526 (20.4%)	
Quartile 3	87 (5.7%)	163 (6.3%)	
Quartile 4	22 (1.4%)	75 (2.9%)	
Weighted Charlson Index	4 (2-6)	4 (3-6)	<0.001
Congestive Heart Failure	1,538 (100%)	2,584 (100%)	1.00
Dementia	127 (8.3%)	156 (6.0%)	0.006
Metastatic Cancer	29 (1.9%)	51 (2.0%)	0.84
Length of Hospitalization (days), median (IQR)	4 (3-7)	5 (3-7)	0.21
Used Intensive Care	354 (23.0%)	592 (22.9%)	0.94
Mortality			
In-hospital	91 (5.9%)	86 (3.3%)	<0.001
30 days	200 (13.0%)	212 (8.2%)	<0.001
90 days	391 (25.4%)	420 (16.3%)	<0.001
1-year	740 (48.1%)	873 (33.8%)	<0.001

Table S3: Characteristics of Pneumonia Patients and Hospitalizations, by BMI

	Normal BMI N=1,736	Overweight or Obese BMI N=2,152	*p*-value
Age (years), median (IQR)	81 (73-87)	77 (70-83)	<0.001
Male, N(%)	838 (48.3%)	1,062 (49.4%)	0.14
Race, N(%)			<0.001
White/Caucasian	1,478 (85.1%)	1,698 (78.9%)	
Black/African American	206 (11.9%)	381 (17.7%)	
Other	52 (3.0%)	73 (3.4%)	
Married/Partnered, N(%)	771 (44.4%)	1,105 (51.4%)	<0.001
Wealth, N(%)			0.02
Net neg. or zero	228 (13.1%)	284 (13.2%)	
Quartile 1	546 (31.5%)	732 (34.0%)	
Quartile 2	399 (23.0%)	538 (25.0%)	
Quartile 3	323 (18.6%)	320 (14.9%)	
Quartile 4	240 (13.8%)	278 (12.9%)	
Income, N(%)			0.002
None	675 (38.9%)	788 (36.6%)	
Quartile 1	574 (33.1%)	711 (33.0%)	
Quartile 2	322 (18.6%)	396 (18.4%)	
Quartile 3	133 (7.7%)	182 (8.5%)	
Quartile 4	32 (1.8%)	75 (3.5%)	
Weighted Charlson Index	3 (1-5)	3 (2-5)	<0.001
Congestive Heart Failure	828 (47.7%)	1,114 (51.8%)	0.01
Dementia	262 (15.1%)	219 (10.2%)	<0.001
Metastatic Cancer	89 (5.1%)	110 (5.1%)	0.98
Length of Hospitalization (days), median (IQR)	5 (3-8)	5 (3-8)	0.58
Used Intensive Care	312 (18.0%)	474 (22.0%)	0.002
Mortality			
In-hospital	208 (12.0%)	194 (9.0%)	0.003
30 days	349 (20.1%)	293 (13.6%)	<0.001
90 days	503 (29.0%)	449 (20.9%)	<0.001
1-year	785 (45.2%)	749 (34.8%)	<0.001

Table S4: Characteristics of AMI Patients and Hospitalizations, by BMI

	Normal BMI N=698	Overweight or Obese BMI N=1,232	*p*-value
Age (years), median (IQR)	80.5 (73-86)	75 (69-82)	<0.001
Male, N(%)	333 (47.7%)	705 (57.2%)	<0.001
Race, N(%)			<0.001
White/Caucasian	617 (88.4%)	994 (80.7%)	
Black/African American	63 (9.0%)	186 (15.1%)	
Other	18 (2.6%)	52 (4.2%)	
Married/Partnered, N(%)	350 (50.1%)	728 (59.1%)	<0.001
Wealth, N(%)			0.17
Net neg. or zero	63 (9.0%)	137 (11.1%)	
Quartile 1	181 (25.9%)	356 (28.9%)	
Quartile 2	163 (23.4%)	283 (23.0%)	
Quartile 3	158 (22.6%)	257 (20.9%)	
Quartile 4	133 (19.1%)	199 (16.2%)	
Income, N(%)			0.001
None	259 (37.1%)	392 (31.8%)	
Quartile 1	239 (34.2%)	370 (30.0%)	
Quartile 2	114 (16.3%)	276 (22.4%)	
Quartile 3	66 (9.5%)	147 (11.9%)	
Quartile 4	20 (2.9%)	47 (3.8%)	
Weighted Charlson Index	3 (2-5)	3 (2-5)	0.04
Congestive Heart Failure	396 (56.7%)	666 (54.0%)	0.27
Dementia	54 (7.7%)	52 (4.2%)	<0.001
Metastatic Cancer	12 (1.7%)	15 (1.2%)	0.36
Length of Hospitalization (days), median (IQR)	5 (3-8)	5 (3-8)	0.52
Used Intensive Care	389 (55.7%)	735 (59.7%)	0.09
Mortality			
In-hospital	72 (10.3%)	110 (8.9%)	0.32
30 days	124 (17.8%)	161 (13.1%)	0.005
90 days	173 (24.8%)	207 (16.8%)	<0.001
1-year	277 (39.7%)	315 (25.6%)	<0.001

Table S5: Numbers and Adjusted Hazards (or Odds) Ratios for Mortality in Primary and Sensitivity Analyses

Analysis	Methodology	CHF		Pneumonia		AMI	
		N	Adjusted HR (95% CI)	N	Adjusted HR (95% CI)	N	Adjusted HR (95% CI)
1-Year Mortality							
Principal	Multivariable Cox Proportional Hazards Model with all hospitalizations and robust standard errors.	4,287	**0.68** (0.59-0.79)	4,182	**0.74** (0.64-0.85)	2,001	**0.65** (0.53-0.80)
Sensitivity #1	Multivariable Cox Proportional Hazards Model with all hospitalizations with stable or increasing weight trajectory and robust standard errors.	3,500	**0.71** (0.60-0.84)	3,402	**0.78** (0.67-0.91)	1,724	**0.62** (0.50-0.78)
Sensitivity #2	Multivariable Cox Proportional Hazards Model with all hospitalizations, adjustment for illness severity, and robust standard errors.	4,287	**0.66** (0.57-0.77)	4,182	**0.72** (0.62-0.83)	2,001	**0.71** (0.57-0.88)
Sensitivity #3	Multivariable Logistic Regression Model of all hospitalizations with robust standard errors.	4,287	**0.60** (0.50-0.73)	4,182	**0.68** (0.58-0.81)	2,001	**0.60** (0.48-0.76)
Sensitivity #4	Multivariable Cox Proportional Hazards Model with one randomly selected hospitalization per person.	2,258	**0.72** (0.63-0.83)	2,933	**0.75** (0.66-0.84)	1,565	**0.60** (0.49-0.72)
Sensitivity #5	Multivariable Cox Proportional Hazards Model among age and sex-matched pairs, with robust standard errors.	2,954	**0.67** (0.57-0.77)	3,120	**0.73** (0.64-0.84)	1,330	**0.66** (0.52-0.84)
Sensitivity #6	Multivariable Cox Proportional Hazards Model with adjustment for functional limitations and co-morbid disease, with robust standard errors.	3,684	**0.67** (0.57-0.78)	3,576	**0.75** (0.64-0.86)	1,627	**0.69** (0.55-0.87)
5-Year Mortality, Conditional on Survival to 1 Year							
Principal	Multivariable Cox Proportional Hazards Model with all hospitalizations and robust standard errors.	2,567	0.98 (0.84-1.14)	2,472	0.92 (0.81-1.05)	1,362	0.90 (0.71-1.13)
Sensitivity #1	Multivariable Cox Proportional Hazards Model with all hospitalizations with stable or increasing weight trajectory and robust standard errors.	2,149	0.95 (0.80-1.13)	2,081	0.89 (0.76-1.03)	1,195	0.95 (0.74-1.23)
Sensitivity #2	Multivariable Cox Proportional Hazards Model with all hospitalizations, adjustment for illness severity, and robust standard errors.	2,567	0.97 (0.83-1.13)	2,472	0.91 (0.80-1.04)	1,362	0.91 (0.72-1.15)

Sensitivity #3	Multivariable Logistic Regression Model of all hospitalizations with robust standard errors.	2,212	1.02 (0.77-1.35)	2,108	0.82 (0.65-1.02)	1,130	0.90 (0.65-1.25)
Sensitivity #4	Multivariable Cox Proportional Hazards Model with one randomly selected hospitalization per person.	1,476	0.96 (0.83-1.11)	1,822	0.91 (0.80-1.05)	1,075	0.85 (0.68-1.06)
Sensitivity #5	Multivariable Cox Proportional Hazards Model among age and sex-matched pairs.	1,554	1.00 (0.86-1.17)	1,756	0.90 (0.78-1.04)	824	0.85 (0.65-1.11)
Sensitivity #6	Multivariable Cox Proportional Hazards Model with adjustment for functional limitations and co-morbid disease, with robust standard errors.	2,177	0.90 (0.76-1.07)	2,076	0.92 (0.79-1.06)	1,111	0.88 (0.68-1.15)

The referent group in BMI 18.5 to <25.0 kg/m^2. All models adjust for age, sex, race, marital status, education, smoking status, admission year, number of hospitalizations, household wealth, and household income. Bolded values are statistically significant, $p<0.05$.

Table S6: Adjusted Hazard Ratios from Primary Analysis, by overweight, obese, and severely obese BMI

Adjusted HR	CHF	Pneumonia	AMI
1-Year Mortality			
Overweight	**0.66** (0.56-0.79)	**0.75** (0.64-0.87)	**0.63** (0.50-0.79)
Obese	**0.71** (0.57-0.89)	**0.75** (0.61-0.93)	**0.67** (0.48-0.94)
Severely obese	**0.70** (0.54-0.91)	**0.65** (0.49-0.86)	0.74 (0.48-1.12)
5-Year Mortality, Conditional on Survival to 1 Year			
Overweight	0.95 (0.80-1.13)	0.94 (0.82-1.09)	0.83 (0.65-1.07)
Obese	1.09 (0.89-1.34)	0.83 (0.67-1.03)	0.94 (0.67-1.31)
Severely obese	0.90 (0.66-1.20)	0.90 (0.68-1.19)	1.30 (0.85-1.99)

The models adjust for age, sex, race, marital status, education, smoking status, admission year, number of hospitalizations, household wealth, and household income. BMI is classified as overweight (≥25.0 to <30.0 kg/m^2), obese (≥30.0 to <35.0 kg/m^2), and severely obese (≥35.0 kg/m^2). The referent group in BMI 18.5 to <25.0 kg/m^2.
Bolded values are statistically significant, $p<0.05$.

Table S7: Adjusted Hazard Ratios from Primary Analysis, with alternate referent groups

Adjusted Hazard Ratios for 1-Year Mortality, with alternate referent groups			
	Primary Analysis: Ref BMI 18.5 to <25.	Sensitivity Analysis: Ref BMI 20.0 to <25.	Sensitivity Analysis: Ref BMI 22.0 to <25.
CHF	**0.68** (0.59, 0.79)	**0.70** (0.60, 0.82)	**0.74** (0.62, 0.88)
Pneumonia	**0.74** (0.64, 0.85)	**0.77** (0.67, 0.89)	0.85 (0.71, 1.00)
AMI	**0.65** (0.53, 0.80)	**0.69** (0.56, 0.86)	**0.67** (0.53, 0.85)
Adjusted Hazard Ratios for 5-Year Mortality, with alternate referent groups			
	Primary Analysis: Ref BMI 18.5 to <25.	Sensitivity Analysis: Ref BMI 20.0 to <25.	Sensitivity Analysis: Ref BMI 22.0 to <25.
CHF	0.98 (0.84, 1.14)	1.03 (0.88, 1.21)	1.09 (0.90, 1.32)
Pneumonia	0.92 (0.81-1.05)	0.98 (0.85, 1.13)	1.02 (0.87, 1.20)
AMI	0.90 (0.71-1.13)	0.89 (0.71, 1.13)	0.88 (0.68, 1.15)

The models adjust for age, sex, race, marital status, education, smoking status, admission year, number of hospitalizations, household wealth, and household income.
Bolded numbers are statistically significant, p<0.05.

References

1. Keenan PS, Normand S-LT, Lin Z, et al. An administrative claims measure suitable for profiling hospital performance on the basis of 30-day all-cause readmission rates among patients with heart failure. Circ Cardiovasc Qual Outcomes 2008;1(1):29–37.
2. Epstein AJ, Rathore SS, Krumholz HM, Volpp KGM. Volume-based referral for cardiovascular procedures in the United States: a cross-sectional regression analysis. BMC Health Serv Res 2005;5:42.
3. Krumholz HM, Wang Y, Mattera JA, et al. An administrative claims model suitable for profiling hospital performance based on 30-day mortality rates among patients with an acute myocardial infarction. Circulation 2006;113(13):1683–92.
4. Lindenauer PK, Lagu T, Shieh MS, Pekow PS, Rothberg MB. Association of diagnostic coding with trends in hospitalizations and mortality of patients with pneumonia, 2003-2009. JAMA 2012;307(13):1405–13.
5. Valley TS, Sjoding MW, Ryan AM, Iwashyna TJ, Cooke CR. Association of Intensive Care Unit Admission With Mortality Among Older Patients With Pneumonia. JAMA 314(12):1272–9.
